# Reference Values and Influencing Factors Analysis for Current Perception Threshold Testing Based on Study of 166 Healthy Chinese

**DOI:** 10.3389/fnins.2018.00014

**Published:** 2018-01-26

**Authors:** Hexiang Yin, Mingsheng Liu, Yicheng Zhu, Liying Cui

**Affiliations:** Department of Neurology, Peking Union Medical College Hospital, Chinese Academy of Medical Sciences, Beijing, China

**Keywords:** sensory nerve fibers, current perception threshold, neuroselective, reference value, age effects, gender effects, occupational effects

## Abstract

The current perception threshold (CPT) is a device which can evaluate different sensory fibers quantitatively through different frequencies of the electrical stimulus and has been applied in clinical practice. Previous studies have implied that CPT values may be affected by age, gender, and other factors, yet not conclusively. The objective of our study is to clarify the influencing factors of CPT values and establish a reference value range. Twenty healthy volunteers recruited publicly and 146 subjects who took CPT tests in the census of the national project cardiovascular and cerebrovascular diseases in rural areas of China from 2013 to 2015 were analyzed. Past medical history and demographic characteristics such as age, gender, and occupation were collected. Each subject was tested on the left index finger (or back of the left hand) and the right hallux. CPT values of 2000, 250, and 5 Hz on both sites were recorded for statistical analysis. Gender differences were shown at 2000 Hz CPT on the back of the hand and hallux (*p* < 0.01), and male subjects had a higher CPT. Age had a positive correlation with 250 Hz CPT on the index finger (*p* < 0.05, *r* = 1.5), 2000 Hz CPT on the back of the hand (*p* < 0.001, *r* = 1.2) and index finger (*p* < 0.05, *r* = 2.5). Manual workers had a higher 250 Hz CPT on the hallux than mental workers (*p* < 0.01). After investigating the impact of different factors on CPT testing, we established the reference value for subjects with different characteristics.

## Introduction

Cutaneous sensory nerve fibers are typically classified into three varieties according to their cross sectional diameters. These classifications are the larger myelinated A-beta(β) fibers (5–15 μm diameter), lightly myelinated A-delta(δ) fibers (1–4 μm diameter) and the unmyelinated C fibers (0.5–1.5 μm diameter). A-β fibers conduct the senses of touch, pressure, and vibration, while both A-δ fibers and C fibers conducting the senses of pain and temperature, and C fibers also serve as post-sympathetic fibers (McGlone and Reilly, 2010). Evaluation of the sensory nerve fiber's function plays a vital role in the diagnosis and follow-up of certain diseases involving the sensory conducting pathways. Different from classical examination tools, such as cotton, pin, and tuning fork, or neurophysiological methods, such as sensory nerve conduction velocity (SCV), sensory nerve action potential (SNAP), and somatosensory evoked potential (SEP), the current perception threshold (CPT) device can document the functioning of the sensory nerve fibers in a quantitative manner and achieve differential neuro-excitatory effects of the sensory fibers depending on the frequency of stimuli (Katims et al., 1986a,b; Baquis et al., 1999). Evidence suggested that stimuli of 2000, 250, and 5 Hz reflected the functions of A-β, A-δ, C fibers, respectively (Félix et al., 2009; Neurotron, 2010). The definition of CPT on certain body parts is the lowest electric stimuli intensity which can be perceived consistantly, therefore the test measures are recorded as current intensity values ranging from 0.01 to 9.99 mA. Results of CPT below the normal range are suspected as hyperesthesia, while those of CPT above the normal range imply hypoesthesia.

The CPT device has been applied in the diagnosis and evaluation of many clinical conditions, of which diabetic neuropathy is the most common (Rendell et al., 1989; Katims et al., 1991; Rendell and Bamisedun, 1992; Lee et al., 1997; Ro et al., 1999; Menkes et al., 2000; Kurozawa and Nasu, 2001; Oishi et al., 2002; Takekuma et al., 2002; Cui et al., 2003; Prendergast et al., 2004; Lander et al., 2007; Putz et al., 2009; Nather et al., 2011; Griffith et al., 2014). However, more than one study has found differences between the reference values of the research subjects and those provided by the manufacturer (Galvão Mde et al., 2005; Quaghebeur and Wyndaele, 2014), and the influence of factors such as age and gender on CPT values has not been illustrated consistently among various studies (Evans et al., 1992; Takekuma et al., 2000; Tseng et al., 2002; Seong et al., 2009). Therefore, the objective of our study was to explore CPT distribution properties of Chinese subjects and to investigate different influencing factors of CPT. Furthermore, we aimed to establish reference value ranges suitable for subjects with different clinical characteristics.

## Materials and methods

### Subjects

The healthy subjects were recruited through two different approaches. The majority came from the census of the national project cardiovascular and cerebrovascular diseases in rural areas of China from 2013 to 2015, and the others were recruited publicly for CPT testing. The inclusion criteria were for individuals having no history or complaints of sensory dysfunction and having a normal physical examination. Exclusion criteria were as follows: a history of diabetes mellitus (DM), consumption of alcohol, central nervous system diseases, cervical or lumbar spondylosis, malignant tumors, usage of vibratory tools, electrical application implants (such as pace maker), and the presence of skin lesions at the testing site.

This study was performed in compliance with the Code of Ethics of the World Medical Association (Declaration of Helsinki) and was approved by the ethics committee of Peking Union Medical College Hospital. All subjects participated voluntarily after being provided information about the testing procedures. Written informed consent was obtained from each subject prior to participation.

### Data collection

#### Clinical data

Before CPT testing, all of the subjects underwent a medical history investigation and physical examination. Information as follows was collected: name, gender, age, occupation, past medical history, personal history including usage of medications and history of contact with toxins.

#### CPT testing

**Figure 1:**
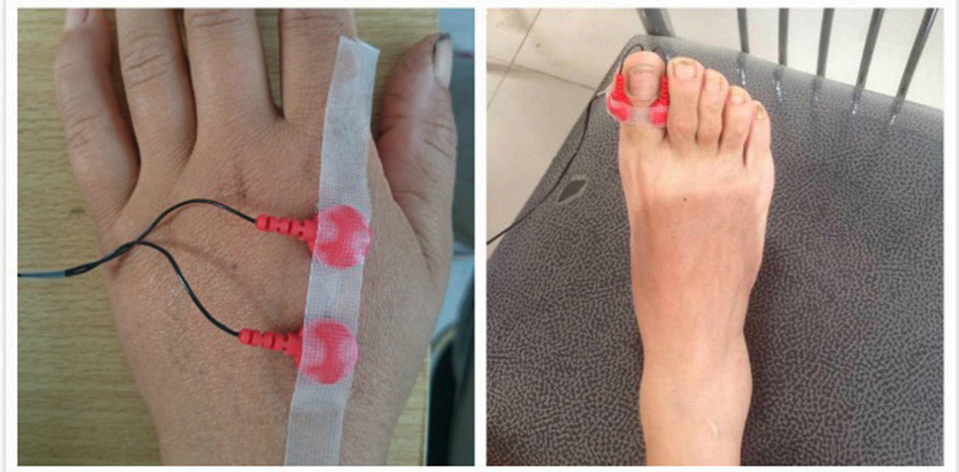
Testing site and the placement of the electrode.

CPT tests were performed with a Neurometer® (Neurotron, Inc., Baltimore, MD, USA) which measures the neuroselective CPTs. The testing sites were selected as either the left index finger or the back of the hand (C7 dermatome) and the right hallux (L4/5 dermatome), and the placement of the electrode is shown in Figure 1. Automatic double-blinded testing was applied. Subjects sat in a quiet room with a comfortable environmental temperature and lighting, and were informed about the procedure carefully beforehand to ensure understanding and better cooperation during the following test. The skin at the testing site was prepared with a skin prep paste and a hypoallergenic electrode gel was applied to the electrodes before placement. The current levels of 2000, 250, and 5 Hz CPTs were tested in turn at each site. The total test time for each subject was no more than 15 min.

### Statistical analysis

The categorical variables were recorded as frequency and percentage. Distribution was evaluated with a normality test (One-Sample Kolmogorov-Smirnov test). Normally distributed variables were presented as the mean (standard deviation) and non-normally distributed variables as the median (interquartile range). Differences between the groups were analyzed with independent sampled *t*-tests for two groups or ANOVA for more than two groups with continuous data and chi square tests for categorical data. Correlation analysis: Pearson analysis was applied to normal distribution while Spearman analysis was applied to non-normal distribution. Clinical factors influencing CPT testing results were accessed by logistic regression analysis. A *p*-value of 0.05 or less was considered statistically significant. Analyses were conducted using SPSS 20.0 (IBM Corp., Armonk, NY, USA).

## Results

### Demographic data

One hundred and sixty-six healthy subjects were included, and the age and gender distribution is shown in Table 1. There is no significant difference in age distribution between the male and female groups (*p* > 0.05). Eight subjects' occupations were not available, with the rest consisting of 107 manual workers and 51 mental workers.

**Table 1:** Age and gender distribution of the 166 subjects.

	***n***	***Mean* ± *SD*, age, y**
Male	37	49.0 ± 13.6
Female	129	52.2 ± 10.0

### CPT data

#### Distribution of CPT data

CPT values of different testing sites are shown in Table 2. A normality test showed all values are normally distributed.

**Table 2:** CPT values of different testing sites [*Mean* ± *SD* (range), unit: 10 μA].

	***n***	**2000 Hz**	**250 Hz**	**5 Hz**
Index finger	35	226.8 ± 63.7 (95–396)	100.0 ± 34.8 (40–192)	57.2 ± 21.8 (13–90)
Hand back	131	136.8 ± 37.8 (46–250)	39.8 ± 11.7 (19–62)	19.9 ± 8.0 (5–41)
Hallux	166	287.7 ± 72.1 (95–492)	124.0 ± 47.0 (10–247)	71.4 ± 34.9 (7–164)

#### Comparison between left and right sides

Of the participants, 34 subjects completed CPT testing on both sides, and the comparisons of the results from both sides at different testing sites are shown in Table 3. There is no significant difference between CPT values obtained from the left and right side, so unilateral data is qualified for formulating the reference values.

**Table 3:** Comparison between values from left and right side (*n* = 34).

**Testing site**	**Frequency (Hz)**	***P*-value**
Hand	2000	0.489
	250	0.508
	5	0.360
Foot	2000	0.415
	250	0.266
	5	0.871

#### Comparison of testing sites

**Figure 2:**
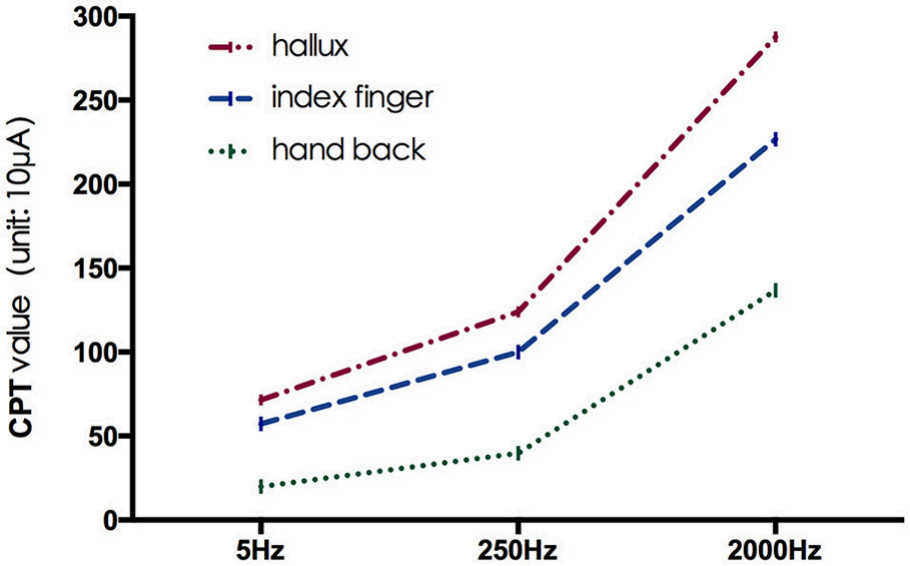
The mean CPT values of the three frequencies at each testing site.

**Figure 3:**
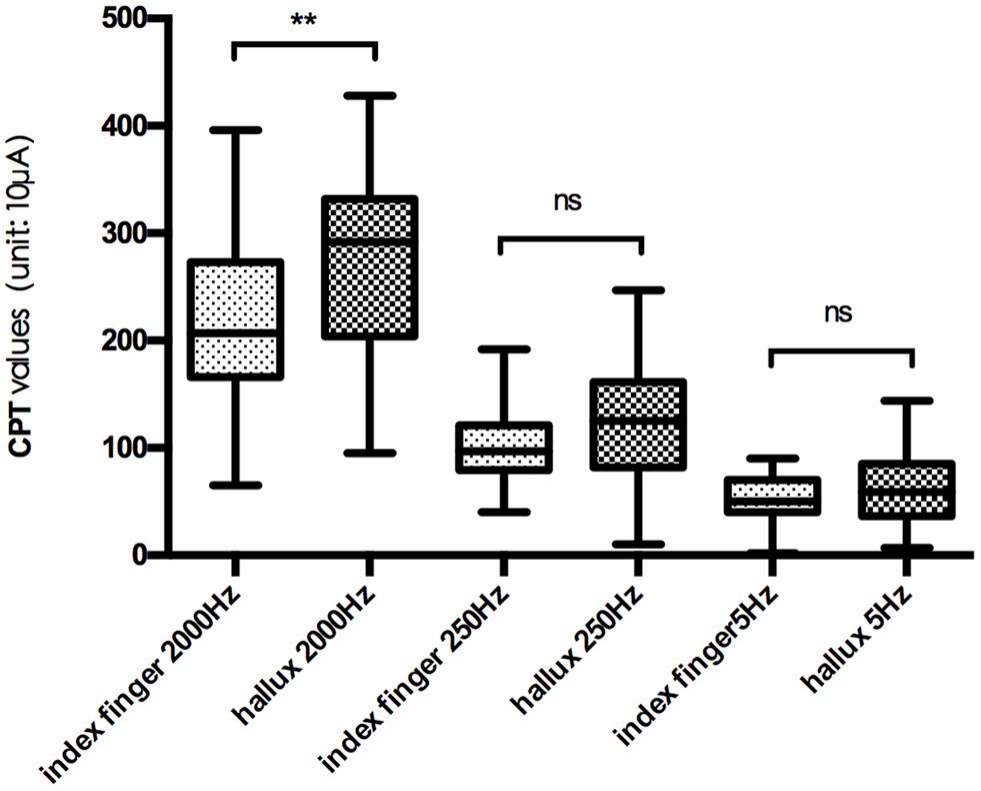
Comparison between CPT values at index finger and hallux. ^**^p < 0.01; ns, no significance.

**Figure 4:**
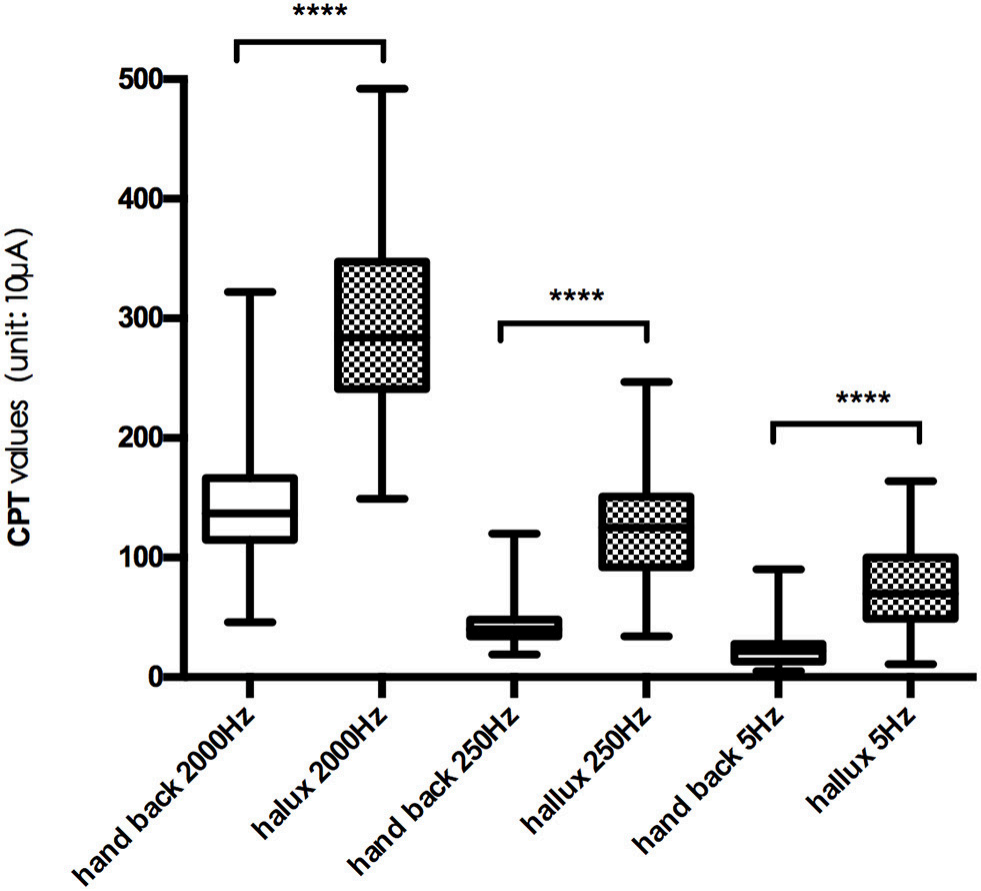
Comparison between CPT values at hand back and hallux. ^****^p < 0.001.

The mean CPT values of the three frequencies at each testing site are shown in Figure 2, and further comparison between CPT values of the same frequency at different testing site are shown in Figures 3, 4, separately. The CPT value for the back of the hand was generally lower than that of the hallux (*p* < 0.001), and a lower CPT value was obtained at the index finger than the hallux under a stimulus of 2000 Hz (*p* < 0.01).

### Influencing factor analysis

Multivariate logistic regression analysis of influencing factors is shown in Table 4. Gender, age and occupation show effects on certain frequencies at different testing sites, respectively. Gender differences were shown at 2000 Hz CPT on the back of the hand and the hallux (*p* < 0.01), and male subjects had a higher CPT. Age had a positive correlation with 250 Hz CPT on the index finger (*p* < 0.05, *r* = 1.5), 2000 Hz CPT on the back of the hand (*p* < 0.001, *r* = 1.2) and the index finger (*p* < 0.05, *r* = 2.5). Manual workers had higher 250 Hz CPT on the hallux than mental workers (*p* < 0.01).

**Table 4:** Multivariate logistic regression analysis of influencing factors (*p-*value).

	**Index finger**			**Hand back**			**Hallux**		
Factors	2000 Hz	250 Hz	5 Hz	2000 Hz	250 Hz	5 Hz	2000 Hz	250 Hz	5 Hz
Gender	0.500	0.756	0.682	0.003	0.612	0.704	0.009	0.721	0.427
Age	0.038 (*r* = 2.5)	0.023 (*r* = 1.5)	0.862	0.000 (*r* = 1.2)	0.214	0.272	0.061	0.928	0.687
Occupation	0.785	0.514	0.986	0.100	0.100	0.363	0.072	0.007	0.426

*r = correlation coefficient*.

### Reference values of CPT testing

Based the results of influencing factor analysis, the reference values of CPT testing for the three frequencies at different sites were established in Table 5. The reference values of 2000 Hz CPT on the hallux were divided by gender, and those of 250 Hz CPT on the hallux were divided by occupation. The reference values of 2000 and 250 Hz CPT on the index finger were established according to age, while those of 2000 Hz CPT on the index finger were divided by age and gender.

**Table 5:** Reference values of CPT testing (unit: 10 μA).

		**Gender/occupation/age (years old)**		***Lower* 95% *CI***	***Mean***	***Upper* 95% *CI***
Index finger (C7, *n* = 35)	2000 Hz	≤50		93.2	209.2	325.2
		>50		111.2	239.2	367.2
	250 Hz	≤50		39.2	87.9	136.7
		>50		32.5	108.1	183.6
	5 Hz	–		14.5	57.2	99.9
Hand back (C7, *n* = 131)	2000 Hz	Male	≤50	65.0	137.3	209.6
			>50	112.8	157.9	203.0
		Female	≤50	37.7	121.0	204.3
			>50	75.0	142.0	209.0
	250 Hz	–		16.9	39.8	62.7
	5 Hz	–		4.2	19.9	35.6
Hallux (L4/5, *n* = 166)	2000 Hz	Male		166.0	304.4	442.8
		Female		141.8	282.9	424.0
	250 Hz	Manual workers		36.2	130.1	224.0
		Mental workers		25.2	107.5	189.8
	5 Hz	–		3.0	71.4	139.8

## Discussion

### Distribution characteristics of normal CPT

Our study shows the distribution characteristics of normal CPT values, which are consistent with previous studies (Katims et al., 1986b; Seong et al., 2009; Neurotron, 2010). The CPT value for the back of the hand was generally lower than that of the hallux (*p* < 0.001), and a lower CPT value was obtained at the index finger than the hallux under a stimulus of 2000 Hz (*p* < 0.01). This could be explained by differences in the density of sensory nerve fibers on different parts of the body (Stevens and Choo, 1996; Johansson et al., 1999; McGlone and Reilly, 2010). Nerve fiber density for human skin appeared to decrease from the trunk to the distal parts of the body, and the intraepidermal nerve fiber density of the distal leg was lower than that of the distal forearm (Besné et al., 2002; Chang et al., 2004).

### Gender effects

Compared with women, men in this study tended to have higher CPT values for the back of the hand and hallux under a stimulus of 2000 Hz. Similarly, Seong et al. (2009) reported gender differences for normal CPT values only appeared in the 2000 Hz stimulus group (*p* < 0.01), and males had a higher perception threshold. Females were reported to have higher myelinated nerve fiber densities than males (Horowitz and Krarup, 1992), which partly gives an explanation on the effects of gender on CPT values under a stimulus of 2000 Hz.

We did not find gender differences at the testing site for the index finger, which is accordant with reports from Quaghebeur et al. (Quaghebeur and Wyndaele, 2014) and Galvão Mde et al. (2005). However, Takekuma et al. revealed gender effects on CPT values for the index finger under stimuli of 250 and 5 Hz in a community-dwelling of 1,000 Japanese, of which males had higher CPT values (Takekuma et al., 2000). The divergence of different studies may result from the sample differences. The number of our subjects was 35, while that of the other two studies were 41 and 101, respectively. Furthermore, there were racial differences found among these studies. Research of intraepidermal nerve fiber (IENF) density in the distal leg was discovered with a significant lower density in men (Goransson et al., 2004), and the majority of IENF was Aδ and C fibers, whose functions could be reflected by CPT values under stimuli of 250 and 5 Hz. Therefore, there are possible gender effects on CPT values under stimuli of 250 and 5 Hz. Whereas, there were no gender differences of IENF shown in other research (McArthur et al., 1998). Moreover, studies of painful sensory neuropathy presented a poor relationship between nerve fiber density and measurements of quantitative sensory testing (Holland et al., 1997; Periquet et al., 1999).

A consistent conclusion has not been drawn for gender effects on CPT values under different stimuli, and studies with larger samples are needed for further exploration.

### Age effects

Age effects were shown in CPT values found at the index finger under stimuli of 250 and 2000 Hz (*p* < 0.05), and the back of the hand under a stimulus of 2000 Hz (*p* < 0.01). Age had a positive correlation with the CPT values referred above. Similar results were revealed in previous studies (Katims et al., 1986b; Takekuma et al., 2000; Costantini et al., 2006; Seong et al., 2009), some of which also found that age had a negative correlation with CPT values under a stimulus of 5 Hz. One study showed no age effects on CPT values in normal subjects or patients of peripheral neuropathy (Evans et al., 1992), while Tseng et al. concluded that age had a positive correlation with CPT values under each frequency of stimuli (Tseng et al., 2002).

The majority of the previous studies report that age has a positive correlation with CPT values under a stimulus 2000 Hz, which is consistent with the phenomenon that sensitivity to vibration and touch decreases in the elderly. The underlying mechanism may lie in age effects on each part of the sensory conducting pathways. It has been proven that both the number of mechanoreceptors and density of nerve fibers in the skin decrease with the increase of age (Cerimele et al., 1990; Gescheider et al., 1994; Besné et al., 2002; Chang et al., 2004; Goransson et al., 2004; Aydog et al., 2006; Vega et al., 2009). Moreover, age-related changes of skin structures such as the soft tissue and the vascular system, which are required to ensure efferent function of the afferent nerve fibers in the skin, play a role in the conduction of sensory information (Bailey, 2001; Farage et al., 2007; Wu et al., 2011; Decorps et al., 2014). A loss of thick myelinated nerve fibers was found with aging, while thin myelinated and unmyelinated nerve fibers were retained (Sato et al., 1985; Drac et al., 1991; Kanda et al., 1991; Verdú et al., 1996; Nakayama et al., 1998; Moriyama et al., 2007). Apart from a loss in quantity, the structural destruction of thick myelinated nerve fibers can exacerbate recession of vibratory senses. It has been proven that the nerve conduction velocity decreases with aging, accompanied by a declining amplitude of SNAP and a prolonged latency time of SEP (Dorfman and Bosley, 1979; Bouche et al., 1993). Furthermore, dramatic age-related differences in the processing of a simple tactile stimulus on the somatosensory network has been revealed (Brodoehl et al., 2013). All of the above factors may lead to an increasing perception threshold in the elderly.

### Occupational effects

In a different approach from the previous normal value studies, we investigate the effects of occupations on CPT values and found that manual workers have higher CPT values at the hallux under stimuli of 250 Hz than that of mental workers (*p* < 0.01). The possible explanation of occupational effects on CPT is the differences in history of contact with risk factors between manual and mental workers. The risk factors which have been reported to be related with changes in perception thresholds, including repetitive movements of arms and hands, and long-term exposure to chemicals, heavy metals and pesticides (Bleecker et al., 2005; Lander et al., 2007; Lubis et al., 2008). The manual workers in our study consisted mainly of farmers, production and transport laborers, and household laborers. The occupation effects are only shown in CPT value of the hallux under 250 Hz, which implies sensory nerve fiber impairment of the distal leg is more severe than the upper extremities and different types of sensory nerve fibers are damaged to a different extent. Without a doubt, more studies of the occupational effects on CPT values are needed to prove its validity.

### Limitations

This was a single source of study in which the majority of the subjects come from countryside of China and females predominate in the study, therefore the representativeness of the study sample is unclear. In addition, though reference values for different conditions have been established based on the impact of subject's characteristics, its sensitivity and specificity for screening and diagnosis of peripheral sensory neuropathy remain to be verified.

## Conclusions

As a neurophysiological tool for quantitative assessment of sensory nerve functions, CPT can selectively test different subtypes of sensory nerve fibers in a quick and non-invasive way. The impact of the different factors on the CPT tests will be revealed as follows. Gender differences were shown at 2000 Hz CPT on the back of the hand and the hallux (*p* < 0.01), and male subjects had a higher CPT. Age had a positive correlation with 250 Hz CPT on the index finger (*p* < 0.05, *r* = 1.5), 2000 Hz CPT on the back of the hand (*p* < 0.001, *r* = 1.2) and the index finger (*p* < 0.05, *r* = 2.5). Manual workers had higher 250Hz CPT on the hallux than mental workers (*p* < 0.01). Reference values for different subject characteristics based on the influencing factor analysis were established.

## Author contributions

HY: conducted the literature search, collected all the data, performed all the data analysis, data interpretation, and wrote the first draft version of the manuscript; ML and YZ: designed the study and standardized testing protocol; ML: supervised analyses, interpreted the data, and reviewed the draft version of the manuscript; LC: designed the study, interpreted the data and reviewed the draft version of the manuscript; All authors read and approved the final manuscript.

### Conflict of interest statement

The authors declare that the research was conducted in the absence of any commercial or financial relationships that could be construed as a potential conflict of interest.
